# Plasma chitotriosidase activity versus CCL18 level for assessing type I Gaucher disease severity: protocol for a systematic review with meta-analysis of individual participant data

**DOI:** 10.1186/s13643-017-0483-x

**Published:** 2017-04-20

**Authors:** Tatiana Raskovalova, Patrick B. Deegan, Ruby Yang, Elena Pavlova, Jérome Stirnemann, José Labarère, Ari Zimran, Pramod K. Mistry, Marc Berger

**Affiliations:** 1 Département d’hématologie biologique, Centre Hospitalier Universitaire Estaing, F-63003 Clermont-Ferrand, France; 2 Laboratoire d’immunologie, Grenoble University Hospital, Grenoble Alpes University, F-38043 Grenoble, France; 3 Lysosomal Disorders Unit, Department of Medicine, Addenbrooke’s Hospital, University of Cambridge, Cambridge, UK; 4 Department of internal medicine, Yale University School of Medicine, New Haven, CT USA; 5 Department of General Internal Medicine, Geneva University Hospital, CH-1211 Geneva, Switzerland; 6 UMR CNRS 5525 TIMC-IMAG, Grenoble Alpes University, F-38043 Grenoble, France; 7 Quality of care unit, CIC 1406 INSERM, Centre Hospitalier Universitaire, CS 10217, 38043 Grenoble Cedex 9, France; 8 Gaucher Clinic, Shaare Zedek Medical Center, Jerusalem, 91031 Israel

**Keywords:** Gaucher disease, Biomarkers, Chemokines, CC, Hexosaminidases, Hepatomegaly, Splenomegaly, Anemia, Thrombocytopenia, Humans

## Abstract

**Background:**

Gaucher disease (GD) is an autosomal recessive lysosomal storage disorder caused by deficiency in acid beta-glucosidase. GD exhibits a wide clinical spectrum of disease severity with an unpredictable natural course. Plasma chitotriosidase activity and CC chemokine ligand 18 (CCL18) have been exchangeably used for monitoring GD activity and response to enzyme replacement therapy in conjunction with clinical assessment. Yet, a large-scale head-to-head comparison of these two biomarkers is currently lacking. We propose a collaborative systematic review with meta-analysis of individual participant data (IPD) to compare the accuracy of plasma chitotriosidase activity and CCL18 in assessing type I (i.e., non-neuropathic) GD severity.

**Methods:**

Eligible studies include cross-sectional, cohort, and randomized controlled studies recording both plasma chitotriosidase activity and CCL18 level at baseline and/or at follow-up in consecutive children or adult patients with type I GD. Pre-specified surrogate outcomes reflecting GD activity include liver and spleen volume, hemoglobin concentration, platelet count, and symptomatic bone events with imaging confirmation. Primary studies will be identified by searching Medline (1995 onwards), EMBASE (1995 onwards), and Cochrane Central Register of Controlled Trials (CENTRAL). Electronic search will be complemented by contacting research groups in order to identify unpublished relevant studies. Where possible, IPD will be extracted from published articles. Corresponding authors will be invited to collaborate by supplying IPD. The methodological quality of retrieved studies will be appraised for each study outcome, using a checklist adapted from the *Quality Assessment of Diagnostic Accuracy Studies*-2 tool. The primary outcome will be a composite of liver volume >1.25 multiple of normal (MN), spleen volume >5 MN, hemoglobin concentration <11 g/dL, or platelet count <100 × 10^9^/L. Effect size estimates for biomarker comparative accuracy in predicting outcomes will be reported as differences in areas under receiver operating characteristic curves along with 95% confidence intervals. Effect size estimates will be reported as (weighted) mean differences along with 95% confidence intervals for each biomarker according to outcomes. IPD meta-analysis will be conducted with both one- and two-stage approaches.

**Discussion:**

Valid and precise accuracy estimates will be derived for CCL18 relative to plasma chitotriosidase activity in discriminating patients according to GD severity.

**Systematic review registration:**

PROSPERO 2015 CRD42015027243

**Electronic supplementary material:**

The online version of this article (doi:10.1186/s13643-017-0483-x) contains supplementary material, which is available to authorized users.

## Background

Gaucher disease (GD) is the most frequent recessively inherited lysosomal storage disorder, with an estimated prevalence of one in 40,000 to 60,000 individuals in the general population [1]. GD is caused by the accumulation of glucosylceramide (glucocerebroside) in tissue macrophages resulting from deficiency in lysosomal acid beta-glucosidase (glucocerebrosidase) activity [2]. Gaucher cells (i.e., glucocerebroside-laden macrophages) displace and/or affect normal cells in bone marrow and visceral organs, causing anemia, thrombopenia, hepatosplenomegaly, skeletal manifestations, and organ dysfunctions [3].

The clinical spectrum of GD ranges from lethal disease during infancy to symptom onset in late adulthood [4]. Although early onset of GD in childhood generally predicts rapidly progressive form, the natural course of the disease is rather unpredictable [4]. Additionally, GD may progress at different rates in different organs and disease severity in one organ may not relate to disease in other organs [4]. Type I GD, which accounts for 90% of all patients with GD in Western countries, is characterized by the absence of early neurological manifestation onset in contrast to the much less frequent acute (type II) and chronic (type III) neuronopathic variants [5].

Since 1991, enzyme replacement therapy has been the standard of care for patients with severe GD [6–8]. Substrate reduction therapy has also proved to be safe and effective in reducing hepatosplenomegaly and improving hematological parameters [7, 8]. Quantitative measurement of surrogate biochemical markers may be useful for assisting clinicians in their decision making for the initiation or adjustment of enzyme replacement or substrate reduction therapy, in patients with GD [9].

Current guidelines recommend measuring plasma chitotriosidase activity for monitoring GD in conjunction with clinical assessments [9]. Chitotriosidase, a human analog from non-vertebrate chitinase, is specifically expressed by chronically activated phagocytes and released by glucocerebroside-laden macrophages [10]. Plasma chitotriosidase activity is elevated 1000-fold above normal values in patients with active GD and has been suggested to indicate total body Gaucher cell load [11]. Plasma chitotriosidase activity level correlates with liver and spleen volume, hemoglobin concentration, platelet count, and bone manifestations [12]. Plasma chitotriosidase activity decreases dramatically after initiation of enzyme replacement therapy and rises when treatment is stopped [13].

Yet monitoring plasma chitotriosidase activity has limitations. First, analysis of plasma chitotriosidase activity is technically complex and not standardized across laboratories [14], rendering its interpretation tricky [15]. Second, 6% of the population are homozygous for a chitotriosidase variant, due to a 24-base pair duplication in the chitotriosidase gene, and are deficient in chitotriosidase activity [16]. Third, 35% of the population are heterozygous for this null chitotriosidase variant and express approximately half the activity observed in those who are wild type [15]. Fourth, other polymorphisms have been reported, leading to slightly impaired enzyme activity compared with wild type [10].

CC chemokine ligand 18 (CCL18), originally named pulmonary and activation-regulated chemokine (PARC), is a CC chemokine produced by macrophages in response to “alternative activation” [15]. Plasma CCL18 level is elevated 10- to 50-fold above normal values in patients with active GD. Plasma CCL18 originates from Gaucher cells disseminated in various body locations and reflects the overall body burden of Gaucher cells [17]. CCL18 level correlates with liver and spleen volume, platelet count, history of osteonecrosis, and number of anatomical sites of osteonecrosis [15, 18]. Despite a strong correlation between the two biomarkers [15], CCL18 has theoretically potential advantages over chitotriosidase for monitoring GD activity. First, enzyme-linked immunosorbent assay (ELISA) of CCL18 is amenable to standardization across laboratories [15]. Second, CCL18 is suitable for measuring GD activity in patients who are deficient in chitotriosidase activity [17]. Third, CCL18 levels tend to reflect more closely spleen volume and platelet count during treatment than does chitotriosidase activity [15].

Although chitotriosidase activity and CCL18 are considered equivalent in routine practice, a large-scale, head-to-head comparison of their performance in assessing GD severity is currently lacking. We hypothesize that research evidence on the comparative accuracy for these two biomarkers can be strengthened by secondary analysis of individual participant data (IPD) from primary studies.

### Aims and objectives

The aim of this systematic review with meta-analysis of IPD is to compare the accuracy of plasma chitotriosidase activity and CCL18 level for assessing type I GD activity. The primary hypothesis guiding this project is that both plasma chitotriosidase activity and CCL18 level relate to visceral, hematological, and skeletal disease activity in GD patients. The secondary hypothesis is that the accuracy of chitotriosidase activity and CCL18 may differ in discriminating GD patients according to disease severity.

The primary objective is to compare the accuracy of CCL18 relative to chitotriosidase activity in discriminating patients with a composite of hepatomegaly, splenomegaly, anemia, or thrombocytopenia. The secondary objective is to compare the accuracy of CCL18 relative to chitotriosidase activity for discriminating patients with symptomatic bone events with imaging confirmation.

## Methods

### Study design

This systematic review with meta-analysis of IPD will be conducted according to current guidelines [19, 20]. The present protocol complies with the *Preferred Reporting Items for Systematic review and Meta-Analysis (PRISMA)*-*Protocol* 2015 statement [21]. It describes how primary studies will be identified, data collection will be undertaken, IPD will be analyzed and findings will be interpreted.

### Eligibility criteria

#### Study designs

Eligible studies include cross-sectional and cohort studies measuring both plasma chitotriosidase activity and serum CCL18 level at baseline and/or at follow-up. Because randomized controlled trials evaluating enzyme replacement or substrate reduction therapy are a special case of prospective cohort studies [22], they will be also considered for inclusion. Yet, diagnostic accuracy or retrospective case-control studies evaluating the performance of chitotriosidase activity and/or CCL18 level in discriminating patients with GD and healthy control subjects, as well as case reports are not within the scope of this review.

#### Participants

Primary studies enrolling consecutive children or adult patients with objective confirmation of GD will be included. We will focus on non-neuronopathic (i.e., type I) GD because this is the most prevalent phenotype with visceral manifestations being improved by enzyme replacement therapy. Naïve participants (i.e., without a history of treatment) as well as those receiving symptomatic treatment or undergoing enzyme replacement or substrate reduction therapy will be considered for inclusion. Deficiency in chitotriosidase activity will not be an exclusion criterion. Studies enrolling less than 10 participants will be excluded from the review.

#### Index test

Relevant methods for quantitative measurement of serum CCL18 level include enzyme-linked immunosorbent assay (ELISA) [17] and dissociation-enhanced lanthanide fluoroimmunoassay (DELFIA) [23].

#### Comparator

The comparator is quantitative measurement of plasma chitiotriosidase activity with the use of fluorogenic substrate molecules, including 4-methyllumbelliferyl-chitobiose, 4-methyllumbelliferyl-chitotriose, or 4-methyllumbelliferyl-deoxy-chitotrioside [11, 14, 24].

#### Outcomes

Pre-specified surrogates reflecting disease severity include anemia, thrombocytopenia, splenomegaly, hepatomegaly, and symptomatic bone events with imaging confirmation [25].

#### Timing and setting

Studies with baseline and/or follow-up data for CCL18 level, chitotriosidase activity, and one or more pre-specified outcomes will be included. Because patients were enrolled at varying time from disease onset across studies, we will collect the age at GD diagnosis and at evaluation. We anticipate that the length of follow-up ranges between 6 and 12 months for most randomized controlled trials. However, longer follow-up times are expected for patients enrolled in observational prospective cohort studies, study extensions, or ongoing registries. There will be no restrictions by type of setting.

#### Language

Electronic literature searches will be conducted without language restrictions. However, full-text articles published in languages other than English, Russian, or French will not be assessed for eligibility because of limited resources for translation. For transparency purpose, we will provide a list of potentially relevant articles published in other languages in an appendix.

### Information sources

#### Electronic databases

Studies will be identified by searching Medline via PubMed (1995 onwards), EMBASE via Ovid (1995 onwards), and Cochrane Central Register of Controlled Trials (CENTRAL) via Wiley interface (current issue). The first report on increased plasma chitotriosidase activity in patients with GD was published in 1994 [11]. The first paper reporting on increased chemokine CCL18/PARC level in Gaucher disease was published by the same research group, 10 years later [17]. Because a direct comparison of the accuracy for these two biomarkers was unlikely to be published before this date, our searches have been restricted to 1995 onwards. Our preliminary searches did not identify any relevant citation prior to 2004.

#### Other information sources

We will supplement the electronic search by scanning the reference lists of retrieved original articles and previously published review articles for additional studies. We will search clinicaltrials.gov and the WHO international clinical trials registry platform in order to identify enzyme replacement and/or substrate reduction therapy trials that routinely evaluated chitotriosidase activity and CCL18 as surrogate biochemical markers of GD activity. Additionally, we will contact research groups, authors of relevant articles, and prominent clinicians within the field in order to identify ongoing or completed but not yet published relevant studies.

### Search strategy

Electronic search strategies will be developed by a member from the coordinating group and critically reviewed by a health sciences librarian, using the *Peer Review of Electronic Search Strategies (PRESS)-2015 Guideline Statement* [26]. The search concepts will include plasma chitotriosidase activity, CCL18, biological markers, enzyme replacement therapy, and Gaucher disease. We will use both standardized medical subject heading (MeSH) and text words (Additional file 1: Appendix 1). No type of document restrictions will be applied and no methodology filters will be used. After the Medline search strategy is finalized, adjustments will be made to account for differences in syntax and subject headings (EMTREE) across electronic bibliographic databases. A member from the coordinating group with experience of searching for systematic reviews will execute electronic searches.

### Study records

#### Data management

Literature search results will be uploaded along with titles and abstracts into a reference management software. We will attempt to identify duplicate publications reporting data from the same study by comparing author names, study sites, and sample sizes. Ultimately, corresponding authors will be contacted to obtain clarification on potential overlapping or inconsistencies across multiple reports of the same study.

#### Selection process

Two review authors will independently screen citation titles and abstracts, where available, yielded by the literature search against pre-specified eligibility criteria. The screening process will assess whether the citation (1) reported data from an original research study, (2) focused on patients with GD, (3) evaluated plasma chitotriosidase activity, (4) evaluated serum CCL18 level, and (5) captured one or more pre-specified outcomes of interest. The two review authors will rate each citation using a “relevant,” “irrelevant,” “unsure” (whether relevant or irrelevant) designation. Full-text articles will be retrieved for citations that received a “relevant” or “unsure” classification from at least one of the two review authors. Records rated “irrelevant” by the two review authors will be discarded for further review.

Two review authors will independently assess full-text articles and decide if they meet pre-specified eligibility criteria, using a standardized eligibility form (Additional file 2: Appendix 2). We will contact corresponding authors to obtain additional information about study eligibility, where necessary. Disagreements will be resolved by discussion between the two review authors. Reasons for excluding study records will be recorded.

#### Data collection

Two review authors will independently perform qualitative and quantitative data extraction. For this purpose, a data extraction form will be developed, pretested, and adjusted as necessary. Disagreements will be resolved by discussion between the two review authors.

Where possible, IPD will be extracted from published articles and entered into a database. A hematologist with expertise in GD research and clinical management will invite corresponding authors and/or principal investigators of eligible primary studies to collaborate in this systematic review project by providing us with IPD. Each corresponding author and/or principal investigator will be first approached during international conferences or contacted by both regular and electronic mails. Contact information will be retrieved from published articles and online search. A reminder will be e-mailed to non-respondents 1 month later. A co-author of the primary study will be contacted in case of non-response from both the principal investigator and corresponding author. Pharmaceutical companies that funded clinical trials of enzyme replacement or substrate reduction therapy will be also contacted.

Correspondence will enclose a cover letter, the systematic review protocol, the list of requested variables, and a consent form for participating in the systematic review [27]. The cover letter will emphasize the collaborative nature of the project, ensure that supplied data will be held securely and treated as confidential, and offer participants the opportunity for co-authoring the final report.

If they agree to participate, investigators will be asked for providing de-identified individual data for all patients enrolled in studies. Ultimately, investigators unable to provide IPD will be invited to provide us with aggregate data in tabular formats for pre-specified analyses. Investigators who decline to provide IPD will be surveyed for identifying potential reasons for not participating in the project.

#### Data checking

For each primary study, the data will be cross-checked with respect to range, missing values, and consistency with published reports. Attempts will be made to resolve inconsistencies by discussion with primary study investigators. If required, converted data will be sent to the investigators for verification. Because the relationship between the biomarkers under review (i.e., plasma chitotriosidase activity and CCL18) and the pre-specified outcomes is observational in nature, we will not assess randomization integrity nor selective outcome reporting for randomized controlled trials of enzyme replacement or substrate reduction therapy. We anticipate that patients who are deficient for chitotriosidase activity at baseline will not be tested for this biomarker at follow-up visits.

Raw data from all primary studies will be collated into a single meta-analysis database, including a primary study identifier. Where possible, participants enrolled in several primary and/or extension studies will be assigned a unique identifier. All primary study raw datasets and meta-analysis databases will be stored in a password-protected area of a secure server. Access to databases will be limited to staff working directly on the project. No data will be used for any other purpose without permission of all collaborators.

### Data items

Details of study- and participant-level characteristics will be extracted from reports of primary published articles at this stage. Requested IPD items include variables collected at baseline and at each follow-up visit (Table 1). Studies with insufficient data on a pre-specified outcome despite contacting the authors will be excluded from the IPD meta-analysis for this outcome only.

**Table 1:** Requested variables

Variable	Description
Gender	Male, female
Age at baseline	Year
Age at GD diagnosis	Year
Site of enrollment	Country
Acid beta-glucosidase genotype	–
Chitotriosidase genotype	Deficient[homozygous]; heterozygous; wild type
Previous history of enzyme replacement therapy at enrollment	Never treated with ERT, no ERT within the previous year, ERT within the previous year
Previous history of substrate reduction therapy at enrollment	Never treated with SRT, no SRT within the previous year, SRT within the previous year
Splenectomy	Yes, no
Time to FU visit	Month (0 for baseline)
Treatment received at FU	Placebo, untreated, imiglucerase, velaglucerase alpha, taliglucerase, miglustat, eliglustat, other
Plasma chitotriosidase activity at FU	nmol/mL/h
Serum CCL18 level at FU	ng/mL
Hemoglobin concentration at FU	g/dL
Platelet count at FU	10^9^/L
White blood cell count at FU	10^9^/L
Liver volume at FU	MN
Spleen volume at FU	MN
Previous history of bone event	Yes, no
Osteonecrosis (i.e., clinical history of bone crises with radiologic or magnetic resonance imaging confirmation) within the previous 12 months	Yes, no
Fracture with imaging confirmation within the previous 12 months	Yes, no
Skeletal site of fracture	Spine, hip, femur, knee, distal to the knee, shoulder, clavicle, humerus, elbow, distal to the elbow, rib, other

Abbreviations: *GD* Gaucher disease, *ERT* enzyme replacement therapy, *FU* follow-up; *MN* multiple of normal, *SRT* substrate reduction therapy

### Outcomes

The pre-specified outcomes are derived from liver volume, spleen volume, hemoglobin concentration, platelet count, and symptomatic bone events with imaging confirmation (Table 2). The primary outcome is a composite of hepatomegaly, splenomegaly, anemia, and thrombocytopenia. The secondary outcomes include symptomatic bone manifestations with imaging confirmation, a composite of massive hepatomegaly or splenomegaly, severe anemia or thrombocytopenia, and individual components of the primary and secondary composite outcomes. Cut-off values for continuous parameter are set according to published guidelines or previous studies [28, 29].

**Table 2:** Pre-specified surrogate outcomes reflecting Gaucher disease activity

Outcomes	Definition^a^
Primary outcome	The primary outcome is a composite of one or more individual components among the following:
	- Liver volume >1.25 MN
	- Spleen volume >5 MN
	- Hemoglobin concentration <11.0 g/dL (<10.0 g/dL for patients 6 to 59 months of age)
	- Platelet count <100 × 10^9^/mL
Secondary outcomes	1. Composite of one or more individual components among the following:
	- Liver volume >2.5 MN
	- Spleen volume >15 MN
	- Hemoglobin concentration <8.0 g/dL (<7.0 g/dL for patients 6 to 59 months of age)
	- Platelet count <50 × 10^9^/mL
	2. Symptomatic bone manifestations with imaging confirmation including the following:
	- Osteonecrosis
	- Fracture

Abbreviation: *MN* multiple of normal

^a^Organ volumes will be expressed as multiples of normal (MN) adjusted for body weight. The normal spleen volume will be computed as 2 mL/kg multiplied by weight in kilograms in patient who did not undergo any splenectomy procedure. The normal liver volume will be computed as 25 mL/kg multiplied by weight in kilograms (see the “Methods” section)

We anticipate that organ volumetric data will be derived by computed tomography, magnetic resonance, or ultrasound imaging, depending on the technology available. Organ volumes will be expressed as multiples of normal (MN) adjusted for body weight. The normal spleen volume will be computed as 2 mL/kg multiplied by weight in kilograms in patient who did not undergo any splenectomy procedure. The normal liver volume will be computed as 25 mL/kg multiplied by weight in kilograms.

### Assessment of methodological quality

Two review authors will independently appraise the methodological quality of included studies for each outcome of interest, using a checklist adapted from the *Quality Assessment of Diagnostic Accuracy Studies* (QUADAS)-2 tool [30]. The QUADAS-2 tool comprises four domains including patient selection, index test, reference standard, and flow and timing. Each domain is evaluated in terms of risk of bias while the first three domains are also assessed with respect to applicability to clinical practice [30]. Disagreement in risk of bias and/or applicability between the two review authors will be resolved by discussion.

### Data synthesis

A statistical analysis plan will be developed by the project statistician with complete details and definitions of all planned analyses. This statistical analysis plan will be reviewed by the study chairman and an independent statistician prior to database lock.

#### Analytical sample

A PRISMA-style flow diagram [31] will illustrate the process of primary study selection from identification to inclusion in the meta-analysis. No primary study will be excluded from meta-analysis based on methodological quality assessment results. Primary studies that did not gather data for a pre-specified outcome will not be included in the meta-analysis for this outcome only.

#### Study characteristics

Information on key primary study and participant characteristics, chitotriosidase activity and CCL18 assays, and study methodological quality will be summarized in separate tables.

#### Effect size estimates

Effect size estimates for biomarker comparative accuracy in predicting outcomes will be reported as differences in areas under receiver operating characteristic (ROC) curves along with 95% confidence intervals. Effect size estimates will be reported as (weighted) mean differences along with standard errors and/or 95% confidence intervals, where appropriate. For highly skewed distributions, we will compute (weighted) mean differences (along with standard error) after logarithm transformation and then derived ratios of geometric means along with 95% confidence intervals [32].

#### Data synthesis

As recommended [33], meta-analysis of IPD will be conducted with both two- and one-stage approaches. In two-stage approach, the first stage implies analyzing IPD within primary studies in order to generate study-level effect size point estimates and variances. In the second stage, point estimates from each primary study are combined using conventional meta-analytical methods. Because aggregate data on the comparative accuracy of chitotriosidase activity and CCL18 in predicting pre-specified outcomes were not reported in most published articles, we anticipate that combination of aggregate data with IPD will not be feasible. This two-stage approach provides summary effect size point estimates along with 95% confidence intervals, which account for between-study heterogeneity. Basically, we will use a DerSimonian and Laird’s random-effects meta-analysis model for combining weighted mean differences in plasma chitotriosidase activity and CCL18 levels (after logarithm transformation, if necessary) for patients with and without each pre-specified outcome [34]. Difference in areas under the ROC curve estimates will be pooled using random-effects meta-analysis models for plasma chitotriosidase activity and serum CCL18 level [35].

One-stage approach synthesizes individual patient data in a single step, using a multilevel mixed-effects regression modeling that accounts for patient clustering within primary studies. This approach allows incorporation of patient-level covariates and exploration of potential interactions involving patient- or study-level covariates. Basically, we will fit three-level model for continuous dependent variables (i.e., plasma chitotriosidase activity or serum CCL18 level), with the three levels being defined by observation, patient, and study [36, 37]. Each pre-specified outcome will be entered as a binary independent variable.

Estimates and paired-comparisons of areas under ROC curves will be derived from nonparametric ROC regression models with bootstrap resampling, which accounts for observation clustering within patients and primary studies [38]. To quantify the incremental value of a dual (i.e., a combination of chitotriosidase activity and CCL18) in comparison with a single (i.e., either chitotriosidase activity or CCL18) biomarker strategy, we will compare areas under ROC curves derived from 3-level logistic regression models. No indirect comparisons will be performed.

#### Missing values

We anticipate missing values for biomarkers (i.e., plasma chitotriosidase activity and CCL18) and outcomes of interest (liver volume, spleen volume (for unsplenectomized participants), hemoglobin level, and platelet count). For transparency purpose, the completeness of IPD will be reported for each variable separately and overall for observations, within each primary study. Complete case analysis, which discards all observations with any missing value, will be undertaken in main analysis for each pre-specified outcome. To assess the robustness of our findings, multiple imputation will be used for replacing missing values. Missing values will be imputed within primary studies in two-stage approach.

#### Investigation of heterogeneity

Between-study heterogeneity will be evaluated graphically by examining forest plots and statistically by using the *I* ^2^ inconsistency index [39]. The *I* ^2^ index provides an estimate of the percentage of total variance across studies due to heterogeneity rather than chance. A *I* ^2^ index of 0% indicates no evidence of heterogeneity, and larger values reflect increasing heterogeneity [39].

#### Subgroup analyses

The following patient- and study-level covariates will be entered in turn into one-stage approach model to examine the sources of heterogeneity in summary estimates: age category (i.e., pediatrics versus adult patient), enzyme replacement therapy within the previous year, and fulfillment of QUADAS-2 criteria [30].

#### Sensitivity analysis

To assess the robustness of our findings, we will conduct sensitivity analysis leaving out one primary study at a time. Additional sensitivity analysis will be performed by (1) substituting splenomegaly to splenectomy for the primary and secondary composite outcomes and (2) excluding patients who are deficient for chitotriosidase activity (i.e., homozygotes).

#### Software

Most data manipulation, figures, and analyses will be documented in Stata programs and performed using Stata 14.0 Special Edition (Stata corp, College Station, TX, USA). Multilevel mixed-effects regression will be developed using Stata’s mixed and melogit commands. Other data preparation and analyses will be performed using Review Manager 5 (Cochrane Collaboration).

### Reporting bias

For each outcome of interest reported by 10 or more studies, evidence of publication bias will be assessed graphically by examining a scatterplot of the effect size estimates from primary studies against the standard error [40]. A symmetrical funnel shape would be consistent with the absence of selective reporting. Asymmetry will be formally evaluated for statistical significance by using Egger’s test [40].

### Quality of evidence

We will assess the quality of evidence for summary estimates using the Grading of Recommendations Assessment, Development and Evaluation (GRADE) rating system based on six factors: study design, limitations (i.e., risk of bias), indirectness of evidence, inconsistency in results across studies, imprecision in summary estimates, and likelihood of publication bias [41].

### Protocol amendments

During the conduct of the meta-analysis, no protocol changes will be made unless new information strongly suggests that such changes would strengthen the scientific validity of the findings. Suggested protocol changes may originate from the primary study investigators who participate in the review. If substantive modifications are necessary that may impact on the review conduct or results, including changes of study objectives, data extraction methods, variable definitions, or significant administrative aspects, they will require a formal amendment to the protocol. Overall, the date, description of changes, and rationale for amendments will be reported in a tabular format. The study chairman will ultimately be responsible for approving, documenting, and implementing amendments.

### Committees

#### Coordinating group

The systematic review coordinating group (TR, JL, MB) is responsible for contacting and liaising with primary study authors, data extraction, checking and analyses, interpretation of findings, and preparing manuscripts for publication.

#### IPD meta-analysis collaborative group

The IPD meta-analysis collaborative group will include the members of coordinating group and a representative from each primary study that provided individual participant data. Clinicians with field expertise may be invited to join the IPD meta-analysis collaborative group. Updated lists of the IPD meta-analysis collaborative group members will be available on the Prospective Registry of Systematic Reviews (PROSPERO) website throughout the conduct of the study. The IPD meta-analysis collaborative group members will be consulted at key stages of the project and given the opportunity to be involved in data analysis, interpretation of the findings, and critical review of the final manuscript.

### Dissemination policy

Efforts will be made to reduce the interval between data extraction completion and the release of the systematic review and meta-analysis results. The members of the coordinating systematic review group will prepare a manuscript reporting the findings from the meta-analysis in accordance with the 2015 PRISMA-IPD statement [31]. The manuscript draft will be circulated for approval by the members of the IPD meta-analysis collaborative group before submission. The anticipated date of completion is March 2018.

## Discussion

Given the prevalence of GD in the general population [5], most published studies that estimated the accuracy of plasma chitotriosidase activity and CCL18 in predicting disease activity are of relatively limited sample size. A valid and reliable head-to-head comparison of these two biomarkers would require a large sample size that can only be supplied by an international prospective study. Yet, such a study is unlikely to be designed due to vested interests, local practices, and scarce resources.

In this context, our meta-analysis provides a unique opportunity to compare the accuracy of CCL18 relative to chitotriosidase activity in discriminating patients according to GD severity. Involving several-fold more participants than single primary studies, meta-analyses can obtain more precise accuracy estimates [42]. Moreover, combining primary studies that enrolled various populations of patients worldwide, meta-analyses produce summary estimates with stronger generalizability [42].

Compared to meta-analysis of aggregate data, our proposed IPD approach has many potential advantages [19]. Indeed, the use of IPD enables implementation of consistent inclusion and exclusion criteria across primary studies, standardization of outcome definitions across primary studies, investigation of heterogeneity in effect size across subgroups of participants, and handling of missing data. Overall, meta-analysis of IPD is considered a more reliable approach than aggregate data meta-analysis [19].

The potential limitations of our IPD meta-analysis should be acknowledged. First, researcher reluctance for sharing raw data is the main obstacle to performing IPD meta-analysis [43]. The potential reasons explaining the lack of data sharing include concerns about patient confidentiality, lack of time, data ownership, the cost for de-identifying and formatting the data, or the availability of the data many years after the completion of the study [43]. Principal investigators for several primary studies have already expressed interest to collaborate in this meta-analysis project and to provide IPD.

Second, the DerSimonian and Laird method may underestimate the true between-study variance although it is widely used in meta-analysis. An overview of published simulations and empirical studies suggested that the estimator proposed by Paule and Mandel may be a better alternative to estimate between-study variance. Yet a simulation study where all concurrent methods would be compared is currently lacking [44].

### Registration

The present protocol has been registered with the international *Prospective Registry of Systematic Reviews* (PROSPERO 2015: CRD42015027243).

## Additional files

Additional file 1: Appendix 1. Literature search strategy for MEDLINE via PubMed. (DOCX 14 kb)

Additional file 2: Appendix 2. Primary study eligibility form. (DOCX 20 kb)

## Abbreviations

CCL18: CC chemokine ligand 18
ELISA: Enzyme-linked immunosorbent assay
GD: Gaucher disease
GRADE: Grading of Recommendations Assessment, Development and Evaluation
IPD: Individual participant data
PRISMA: Preferred Reporting Items for Systematic review and Meta-Analysis
PROSPERO: Prospective Registry of Systematic Reviews
QUADAS: Quality Assessment of Diagnostic Accuracy Studies
ROC: Receiver operating characteristic
