# Supplementary material for: Plasma chitotriosidase activity versus CCL18 level for assessing type I Gaucher disease severity: protocol for a systematic review with meta-analysis of individual participant data
Source: Syst Rev. 2017 Apr 20;6:87. doi: 10.1186/s13643-017-0483-x (PMC5397740; doi:10.1186/s13643-017-0483-x)
Supplement: Supplementary file 1 — Appendix 1. Literature search strategy for MEDLINE via PubMed. (DOCX 14 kb) [file 13643_2017_483_MOESM1_ESM.docx]

**Additional file 1: Appendix 1. Literature search strategy for MEDLINE via Pubmed.**

Raskovalova T et al. Plasma Chitotriosidase Activity versus Serum CCL18 Level for Assessing Type I Gaucher Disease Severity. Protocol for a Systematic Review with Meta-analysis of Individual Participant Data.

Date range: from January 1995 to July 2015, limited to Humans

Date searched: 2015.07.23

| #1 | Chitotriosidase[Supplementary Concept] OR chitotriosidase[Text Word] | 354 |
| --- | --- | --- |
| #2 | CCL18 protein, human[Supplementary Concept] OR CCL18[Text Word] | 305 |
| #3 | Biological markers[MeSH] OR biomarker[Text Word] OR marker[Text Word] | 541,913 |
| #4 | #1 OR #2 OR #3 | 542,248 |
| #5 | Enzyme replacement therapy[MeSH] OR enzyme replac*[Text Word] | 2,676 |
| #6 | (Substrate[Text Word] AND reduc*[Text Word]) OR substrate depriv*[Text Word] | 15,378 |
| #7 | Miglustat[Supplementary Concept] OR miglustat[Text Word] OR Zavesca[Text Word] | 238 |
| #8 | Eliglustat[Supplementary Concept] OR eliglustat[Text Word] | 17 |
| #9 | Imiglucerase[Supplementary Concept] OR imiglucerase[Text Word] OR Cerezyme[Text Word] | 293 |
| #10 | Velaglucerase alfa, human[Supplementary Concept] OR velaglucerase [Text Word] OR vpriv[Text Word] | 40 |
| #11 | Taliglucerase alfa[Supplementary Concept] OR taliglucerase[Text Word] OR elelyso[Text Word] | 15 |
| #12 | #5 OR #6 OR #7 OR #8 OR#9 OR #10 OR #11 | 18,139 |
| #13 | #4 OR #12 | 559,080 |
| #14 | Gaucher disease[MeSH] OR Gaucher[Text Word] | 2,359 |
| #15 | #13 AND #14 | 979 |
