# Supplementary material for: Plasma chitotriosidase activity versus CCL18 level for assessing type I Gaucher disease severity: protocol for a systematic review with meta-analysis of individual participant data
Source: Syst Rev. 2017 Apr 20;6:87. doi: 10.1186/s13643-017-0483-x (PMC5397740; doi:10.1186/s13643-017-0483-x)
Supplement: Supplementary file 2 — Appendix 2. Primary study eligibility form. (DOCX 20 kb) [file 13643_2017_483_MOESM2_ESM.docx]

### Additional file 2: Appendix 2. Primary study eligibility form.

Raskovalova T et al. Plasma Chitotriosidase Activity versus Serum CCL18 Level for Assessing Type I Gaucher Disease Severity. Protocol for a Systematic Review with Meta-analysis of Individual Participant Data.

| REF ID |  | Reviewer |  | Date |  |
| --- | --- | --- | --- | --- | --- |
| Author, year | |  | | | |
| Journal / Source | |  | | | |
| Study ID registration | |  | | Not reported | |
| Citation | |  | | | |
| Publication type | | Full text  Abstract  Other , specify: | | | |
| Other relevant publications | | No  Yes (please list in page 2) | | | |
| Fate | | Included  Decision pending  Check references  Use for discussion  Excluded with listing  Excluded without listing | | | |

| **Eligibility assessment** | | | |
| --- | --- | --- | --- |
| **Type of study** | | | |
| Is the study described as primary research?  (cross-sectional study, cohort study, or RCT) | Yes | No | Unclear |
| **Participants** | | | |
| Were the participants diagnosed as patients with Gaucher disease? | Yes | No | Unclear |
| **Chitotriosidase** | | | |
| Was plasma chitotriosidase activity measured? | Yes | No | Unclear |
| **CCL18** | | | |
| Was serum CCL18 level measured? | Yes | No | Unclear |
| **Outcomes** | | | |
| Did the study report any of prespecified outcomes?  (anemia, thrombocytopenia, hepatomegaly, splenomegaly, symptomatic bone events) | Yes | No | Unclear |
| **Other** | | | |
| Is the study a duplicate publication? | Yes | No | Unclear |
| **Final decision** | Include | Exclude | Unclear |

| **Other relevant citations** | |
| --- | --- |
| **REF ID** | **Citation** |
|  |  |
|  |  |
|  |  |
|  |  |
|  |  |
|  |  |
